# Evaluation of the Structural, Physicochemical and Functional Properties of Dietary Fiber Extracted from Newhall Navel Orange By-Products

**DOI:** 10.3390/foods10112772

**Published:** 2021-11-11

**Authors:** Jiaqi Sang, Lu Li, Jing Wen, Qingqing Gu, Jijun Wu, Yuanshan Yu, Yujuan Xu, Manqin Fu, Xian Lin

**Affiliations:** 1 Sericulture and Agri-Food Research Institute, Guangdong Academy of Agricultural Sciences/Key Laboratory of Functional Foods, Ministry of Agriculture/Guangdong Key Laboratory of Agricultural Products Processing, Guangzhou 510610, China; sjq05176729@163.com (J.S.); lilu045@163.com (L.L.); wujijun@gdaas.cn (J.W.); yuyuanshan@gdaas.cn (Y.Y.); guoshuxuyujuan@163.com (Y.X.); fumanqin@gdaas.cn (M.F.); linxian@gdaas.cn (X.L.); 2 College of Agronomy, Jiangxi Agricultural University, Nanchang 330045, China; qingqinggu2006@126.com

**Keywords:** Newhall navel orange, dietary fiber, structural properties, functional properties

## Abstract

Ultrasound-assisted enzymatic treatment was used to treat Newhall navel orange peel and residue, and then the structural, physicochemical and functional properties of extracted soluble dietary fibers (SDF) and insoluble dietary fibers (IDF) were investigated. The structural properties were determined using scanning electron microscopy, X-ray diffraction, FT-IR and monosaccharide composition. Among these dietary fibers, residue-SDF showed a more complex structure, while peel-IDF exhibited a looser structure. Four samples showed representative infrared spectral features of polysaccharides, typical cellulose crystalline structure and diverse monosaccharide composition. Furthermore, residue-IDF exhibited higher oil-holding capacity (2.08 g/g), water-holding capacity (13.43 g/g) and nitrite ion adsorption capacity (NIAC) than other three samples, and residue-SDF showed the highest swelling capacity (23.33 mL/g), cation exchange capacity (0.89 mmol/g) and cholesterol adsorption capacity (CAC) among these dietary fibers. In summary, this study suggests that the residue-SDF and residue-IDF could be used as the ideal dietary fibers for application in the functional food industry.

## 1. Introduction

Orange (*Citrus* L.) fruit is one of the most important fruits in the world. Newhall navel is the main variety of navel orange, which is famous for its large seedless fruit, thin skin, bright color and fragrant, sweet taste. Because of its high vitamin content and rich aroma characteristics, it is well-received by consumers. In addition to a small number of oranges for processing, most oranges are used for marketing fresh. Moreover, a large number of by-products are formed during processing and daily consumption [1], such as peel and residue, accounting for 20~35% of the whole fruit mass. The by-products of orange have been mostly utilized as molasses for animal feed, pectin and fuel production [2,3]. Peel and residue are main by-products of orange, which are rich in dietary fiber (DF). In order to better use the by-products of Newhall navel orange, it is necessary to investigate the structural, physicochemical and functional properties of dietary fiber exacted from Newhall navel orange peel and residue.

Nowadays, DF has attracted wide attention from researchers, which due to its functions of improving human nutritional status, regulating bodily functions, removing heavy metal ions and inhibiting antioxidant activity [4]. Total dietary fiber (TDF) is usually divided into insoluble (IDF) and soluble (SDF) dietary fiber based on their solubility. Meanwhile, IDF and SDF have different physiological functions. IDF, which accounts for 2/3 of natural fiber, has the effect of accelerating fecal excretion and high viscosity. SDF can be used as a therapeutic agent to reduce blood fat and blood sugar levels [5]. A previous study showed that the water-soluble fraction contained 30–50% of the total dietary fiber, which could not only balance dietary fiber intake but also replenish the essential nutrient element of the human body [6]. Compared with cereals, the DF of orange peel contains higher SDF, which has attracted the attention of researchers. Furthermore, the insoluble fibers of Liucheng orange peel had water-holding capacities and oil-holding capacities [2]. Moreover, the water swelling capacity of citrus fiber treated with chemo-mechanical was significantly higher than that of commercial fiber [7], and citrus fiber could also improve the water-binding ability of low-fat frankfurters [8]. Although the effects on the dietary fiber of [9] citrus have been widely studied, the dietary fiber and its functional characteristics of Newhall orange by-products have few reports.

At present, the methods of extracting DF can be divided into enzymatic method, chemical method, ultrasound, wet milling, microbiological methods and microwave treatment [9,10]. The enzymatic method uses two or more enzymes to destroy the cell wall, thereby removing impurities, such as proteins, starches and soluble sugars [9,11], which has the advantage of security, high efficiency and low consumption of energy. Ultrasonic treatment can result in plant cell wall cavitation in a liquid medium, which can increase the yield or rate of extraction as well as reduce extraction time [12]. The chemical method can destroy the cellulose and hemicellulose in the cell wall, thereby altering the morphology of the cell wall [13]. In previous studies, the combination of two different extraction methods is better than the single extraction method. Ultrasound-assisted enzymatic hydrolysis has the advantages of mild extraction conditions, low investment cost and low energy requirements [14]. Yang found the pectin yield attained with the combined enzymatic and ultrasonic extraction was higher than enzymatic extraction, ultrasonic extraction and acidic extraction [15]. Moczkowska found enzymatic-ultrasonic had the highest SDF content and apparent viscosity of flaxseed [16]. However, the preparation of DF extracted from Newhall navel orange by-products by ultrasonic-assisted enzymatic hydrolysis has rarely been reported. Thus, the purpose of this study was to evaluate the effects of an ultrasound-assisted complex enzyme method on the structural, physicochemical and functional properties of DFs extracted from Newhall navel orange peel and residue, which could provide insight into studies on Newhall orange DF and offer a theoretical basis for comprehensive utilization.

## 2. Materials and Methods

### 2.1. Materials and Chemicals

Fresh oranges (*Citrus sinensis* (L.) *Osbeck*) were harvested and processed in Yichang city of Hubei province of China in July 2020. Glycosylase (100 KU/g), neutral protease (100 KU/g) and α-amylase (4000 U/g, Termamyl) were provided by Shanghai Yuanye biotechnology Co., Ltd. (Shanghai, China). Sodium hydroxide (NaOH), disodium hydrogen phosphate dodecahydrate (Na_2_HPO_4_·12H_2_O) and methanol were HPLC grade. Other chemicals utilized were of analytical grade. 

### 2.2. Preparation of Citrus Dietary Fiber

#### 2.2.1. Preliminary Treatment of Orange

The orange fruits were divided into the peel and the residue after extraction of juice. Then, the peel and residue were dried in an air-oven at 50 °C for 24 h. Finally, the dried samples were ground and sieved (80 μm) to obtain peel and residue powders.

#### 2.2.2. Extraction of IDFs and SDFs

The extraction method of IDF and SDF was according to a method with slight modification [17]. An amount of 10 g of peel and residue powders was suspended in water with a material-liquid ratio of 1:20 (*w*/*v*), and their pH was adjusted to 7.0; then, the mixture was treated with ultrasound for 30 min, 40 °C, 400 W. Hence, 1.5% mixed enzyme (α-amylase-glycosylase enzyme 1:1, m/m) was put into the above mixture, followed by 1 h incubation on the 70 °C water bath. The resulting slurries were heated at 100 °C for 10 min to terminate the enzymatic reaction and allowed to cool to room temperature. After that, the suspension was mixed with protease (500 μL) in a 40 °C water bath for 1 h. The resulting slurries were heated at 100 °C for 5 min to terminate the enzymatic reaction, allowed to cool to room temperature and centrifuged at 5000 rpm for 10 min. The resulting precipitate was freeze-dried to obtain the P-IDF and R-IDF. Meanwhile, while the supernatants were collected and added to 95% ethanol (four-fold volumes) for 24 h to collect residues. The residues were freeze-dried to obtain the P-SDF and R-SDF.

### 2.3. Molecular Weight of IDFs and SDFs

According to a previous study [18], the molecular weight of DF was determined by high performance liquid chromatography equipped with a refractive index detector (RI-10A; Shimadzu Corp., Tokyo, Japan) and a BRT105-104-102 column (8 mm × 300 mm). The mobile phase was 0.05 M NaCl solution and was eluted at 0.6 mL/min for 60 min. The column temperature was 40 °C, and the injection volume was 20 μL. The SDFs and IDFs sample was formulated into aqueous solution (5 mg/mL) and filtered through a 0.22 μm filter. A standard dextran curve was prepared, and the molecular weight of SDFs and IDFs was calculated.

### 2.4. Monosaccharide Compositions of IDFs and SDFs

The monosaccharide composition of the samples was detected by HPLC [5]. Each 0.01 g IDF or SDF sample was added into 2 mL 2 mol/L trifluoroacetic acid, hydrolyzed at 100 °C for 8 h, dried trifluoroacetic acid, washed with 1 mL methanol and then added to 1 mL distilled water to dissolve. Then, the hydrolytes of SDF and IDF were derivatized with 0.5 mol/L PMP-methanol solution and 0.3 mol/L NaOH for 1 h at 70 °C. After cooling at ambient temperature, the reacted product was neutralized with 300 μL 0.3 mol/L HCl and 1 mL chloroform. Then, it underwent 10 min of centrifugation at 4800 rpm. Once the supernatant was absorbed, then 1 mL chloroform was added, and the extraction was repeated three times, with the last supernatant filtered through a 0.22 μm membrane. The injection volume was 20 μL. Acetonitrile and 0.1 mol/L phosphate buffer (pH = 6.7) were used as mobile phases A and B at a ratio of 18:82. The flow rate was 1 mL/min.

### 2.5. Scanning Electron Microscopy (SEM)

The micrograph of IDFs and SDFs were observed by scanning electron microscopy SEM (EVO 18, ZEISS, Karlsruhe, Germany). The IDFs and SDFs were placed on a sample holder with double-sided scotch tape and sputter with gold. Subsequently, each sample was transferred to the scanning electron microscope with 800× and 2000× magnification and an acceleration voltage of 15.0 kV.

### 2.6. Fourier Transfer-Infrared Spectrometry (FT-IR)

The FT-IR spectrum of samples was performed in a total reflection Fourier Transform Infrared (ATR-FTIR) instrument (VERTEX 33, Bruker Co. Ltd., Karlsruhe, Germany). The dry powder sample was mixed with potassium bromide (KBr) powder (1:100, *v*/*v*) and pressed into particles for spectrometric measurement. The spectra were measured in the frequency range of 4500~500 cm^−1^ with a resolution of 4 cm^−1^.

### 2.7. X-ray Diffraction (XRD)

The crystalline structure of IDFs and SDFs was analyzed by X-ray polycrystalline (D8 Advance, Bruker Corp., Karlsruhe, Germany) diffractometer at the working voltage of 40 kV and an incident current of 40 mA. The scanning region of the diffraction Angle (2θ) was 3–55°, and the scanning velocity was 2°/min [19]. The relative degree of crystalline (%) was calculated using MDI Jade 6.5 software (Materials Data, Inc., Livermore, CA, USA) using the following equation:

$$\mathrm{DC}\left(\%\right)=A_{c}\times 100/\left(A_{c}+A_{a}\right)$$

where DC is the degree of crystallinity, and A_c_ and A_a_ represent the crystalline and the amorphous area on the X-ray diffractogram, respectively.

### 2.8. Physicochemical and Functional Properties of Orange DF

#### 2.8.1. Water-Holding Capacity (WHC)

Water-holding capacity (WHC) was conducted according to the method described in [19]. First, we added 0.5 g of IDF or SDF sample to 25 mL of distilled water at room temperature (25 °C) for 1 h. After centrifugation at 4800 rpm for 10 min, the residues were extracted weighed. Finally, WHC was determined by the following equation:

$$\mathrm{WHC}\left(g/g\right)=\frac{W_{1}-W_{2}}{W_{2}}$$

where W_1_ and W_2_ are the weights of wet and dry sample, respectively.

#### 2.8.2. Oil-Holding Capacity (OHC)

The OHC was determined using a method modified from Liu et al. [20], adding 1.0 g of IDF or SDF sample to 25 mL of soybean oil for 2 h. After centrifugation at 4800 rpm for 10 min, the precipitate was extracted and then weighed. The OHC was determined according to the following equation.

$$\mathrm{OHC}\left(g/g\right)=\frac{W_{1}-W_{2}}{W_{2}}$$

where W_1_ and W_2_ are the weights of wet and dry sample, respectively.

#### 2.8.3. The Swelling Capacity (SC)

The SC was determined using a method modified from Zhang et al. [21]. An accurately weighed dry sample of 0.3 g was placed in a test tube, the original volume was recorded, and 5 mL of water was added. It was hydrated for 24 h at 25 °C, and the final volume of the swollen fiber was recorded. The SC was calculated using the following equation:

$$\mathrm{SC}\left(\mathrm{mL}/g\right)=\frac{V_{1}-V_{2}}{M}$$

where V_2_ is the volume of the dried sample, V_1_ is the volume of the hydrated sample and M is the weight of the sample.

#### 2.8.4. Cation Exchange Capacity (CEC)

The CEC was determined using a method modified from He et al. [22] with slight modifications. It began with 0.50 g of the sample and 15 mL of 0.1 mol/L HCl, stirred thoroughly and standing at room temperature for 24 h, then filtered with filter paper and repeatedly washed with distilled water to move acid. The residue was added to a conical flask with 100 mL of 15% (*w*/*v*) NaCl with constant stirring. Distilled water was used as a blank sample, titrated with a 0.01 mol/L NaOH solution, and the volume of consumed NaOH solution was recorded. The cation exchange capacity was calculated as follows:

$$\mathrm{CEC}\left(\mathrm{mmol}/g\right)=\frac{\left(V_{1}-V_{0}\right)\times 0.1}{m}$$

where V_1_ is the titrated volume of NaOH of the sample, V_0_ is the titrated volume of NaOH of the blank, 0.1 is the concentration of NaOH, and m is the dry weight of the sample.

#### 2.8.5. Cholesterol Adsorption Capacity (CAC)

CAC was determined using a method modified from Zhang et al. [23]. The yolk was diluted with 9 volumes of distilled water. A total of 1.0 g of the IDF or SDF sample was mixed with 50 mL diluted yolk. The mixture was adjusted to pH 7.0 or 2.0 and incubated in a shaking water bath at 37 °C for 10 min, and the 0.02 mL supernatant was absorbed. The color was developed by adding 0.1 mL of glacial acetic acid reagent and 2 mL of H_2_SO_4_. The absorbance of the sample reagent was measured at 550 nm. According to the standard curve, the corresponding cholesterol content and cholesterol adsorption were determined.

$$\mathrm{CAC}\left(\mathrm{mg}/g\right)=\frac{M_{1}-M_{2}}{W}$$

where M_1_—cholesterol content before adsorption; M_2_—cholesterol content after adsorption; W—IDF and SDF quality.

#### 2.8.6. Nitrite Ion Absorption Capacity (NIAC)

NIAC was determined using a method modified from Zhu et al. [5] with slight modification. A dried sample (1.0 g) was mixed with 100 mL 250 μmol/L NaNO_2_ solution in a conical flask. After centrifugation at 4800 rpm for 5 min, 5 mL of the supernatant was transferred to a 25 mL glass tube and 2 mL of p-aminobenzenesulfonic acid (4 μg/mL) and 1 mL of naphthalenediamine hydrochloride (2 μg/mL) were added to the mixture. The concentration of NaNO_2_ was measured with a UV-1800 spectrophotometer (Shimadzu Corp., Tokyo, Japan) at 538 nm and quantified according to the standard curve.

$$\mathrm{NIAC}\left(\mu g/g\right)=\frac{M_{1}-M_{2}}{W}$$

where M_1_—nitrite content before adsorption; M_2_—nitrite content after adsorption; W—IDF and SDF quality.

### 2.9. Statistical Analysis

All analyses were conducted in triplicate, and the results are expressed as mean standard deviation. One-way analysis of variance (ANOVA) was performed using SPSS 24.0 statistical software (SPSS Inc., Chicago, IL, USA).

## 3. Results

### 3.1. The Extraction Yield of SDFs and IDFs

The SDF and IDF of Newhall navel orange peel were 9.29% and 70.91%, respectively. Compared with SDF and IDF of Newhall navel orange peel, the Newhall navel orange residue had the higher SDF content (15.05%) and IDF content (79.10%), which may be attributed to the disruption of the glycosidic linkages in dietary fiber under ultrasound, thus causing in orange peel a loss of SDF, IDF, hemicellulose and cellulose [24].

### 3.2. Molecular Weight

The HPGPC results showed that there were many small peaks in R-SDF with the main peak having 49.79% peak area, while there were only two small peaks and one main peak of P-SDF with 89.47% peak area. Moreover, there were many small peaks with one main peak in R-IDF with the main peak having 81.87% peak area, while there were only three small peaks of P-SDF with one main peak of 61.66% peak area. According to the retention time and the dextran standard carve, the Mw of four samples was R-SDF (1.07 × 10^6^ Da), R-IDF (1.57 × 10^6^ Da), P-SDF (0.57 × 10^6^ Da) and P-IDF (0.56 × 10^6^ Da), respectively. It could be found that DF of residue possessed higher molecular weight than DF of peel, which indicated that the molecular weight of DF in orange peel was significantly affected by ultrasound-assisted enzymatic method. It might be explained by the fact that ultrasound and enzymatic hydrolysis could destroy the skeleton of DF of peel and convert large molecules into small molecules [18].

### 3.3. The Monosaccharide Composition of SDFs and IDFs

In order to facilitate better exploration of the properties of dietary fiber, the monosaccharide components of four samples were analyzed. Table 1 showed the monosaccharide composition of different DF samples. All the DF samples contained seven monosaccharides, including mannose, rhamnose, galacturonic acid, glucose, xylose, galactose and arabinose. Glucose was the major monosaccharide, followed by galactose and arabinose. This result indicated that the DFs were mainly composed of glucose in cellulose, xylose and arabinose in hemicellulose. Glucose is mainly derived from starch and cellulose, and the glucose contents of P-SDF and R-SDF were relatively high, which might be because ultrasound promoted the hydrolysis of cellulose in the cell wall [25]. Moreover, galacturonic acid and rhamnose are constituents of pectin [25], and galacturonic acid contents of R-SDF and P-SDF were at high levels along with the relatively high content of rhamnose in P-IDF and P-SDF. Therefore, our analysis shows that pectin is the main component of SDF extracted from orange peel. These were the relatively high contents of arabinose and galactose in R-IDF, which indicated that most of the cellulose has been degraded, and part of the hemicellulose has been released. The high content of xylose and galactose in P-IDF indicated that these sugars were important components of orange cell wall. The contents of mannose in these four samples were very low, but mannose can be used to synthesize glycoprotein, which can participate in the immune regulation of the body and has a good effect on the health of the body.

### 3.4. SEM Analyses of SDFs and IDFs

**Figure 1:**
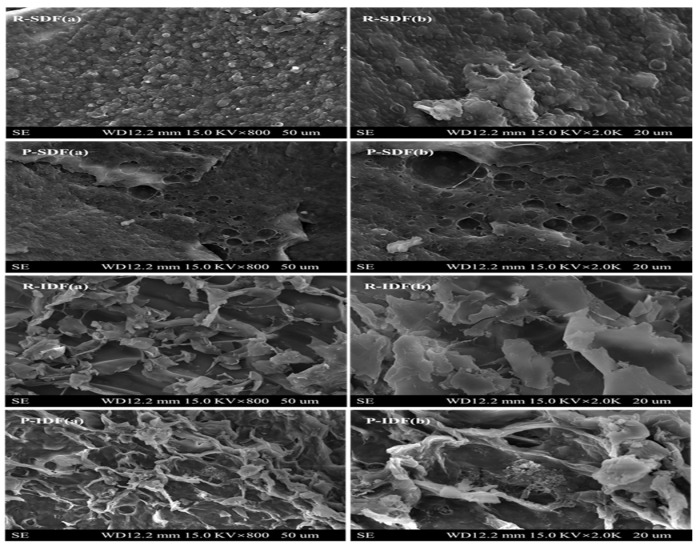
SEM images for R-SDF (**a**,**b**), P-SDF (**a**,**b**), R-IDF (**a**,**b**) and P-IDF (**a**,**b**); (**a**,**b**) represent the micrographs taken at the small and large magnification, respectively. R-SDF represents the residue soluble fiber; P-SDF represents the peel soluble fiber; R-IDF represents the residue insoluble fiber; P-IDF represents the peel insoluble fiber.

In this study, SEM was carried out to investigate the microstructures of IDFs and SDFs extracted from Newhall navel orange peel and residue. As shown in Figure 1, R-SDF had a block shape and a wrinkled surface with a compact texture. In addition, the surface of the R-SDF presented a dense structure, covered with many spherical particles and massive materials, most of which were starch granules and protein wedges [20,26]. P-IDF had looser and more complicated structures, while R-IDF had more wave drapes; this was possibly due to ultrasound treatment having potent oxidation during the extraction process, which could enlarge its surface area. Many voids were formed on the surface of SDFs, which was attributed to enzymes working deep into the fiber molecules, loosening the tightly packed structure of cellulose molecules. However, the spatial network structure of the IDFs was very loose, with large pores and more prominences on the surface. DF with a looser spatial structure had a higher specific surface area, which might affect its adsorption capacities of water, oil and nitrite ion [25].

### 3.5. FT-IR

**Figure 2:**
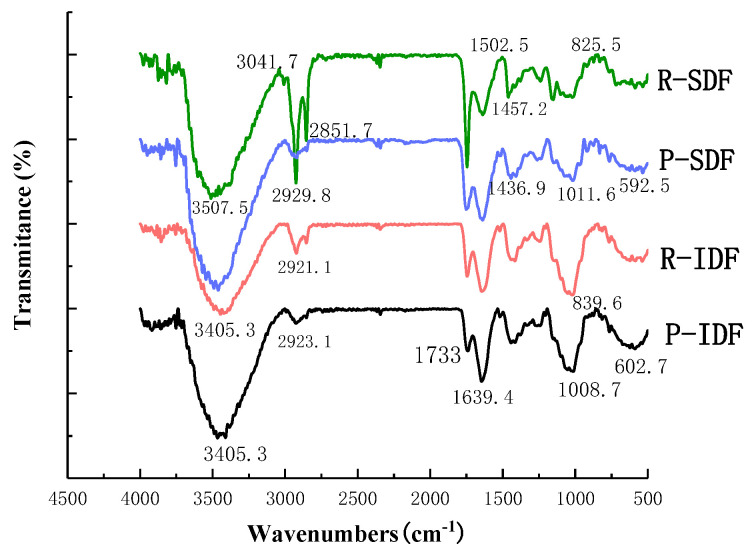
FT-IR spectra for IDFs and SDFs. R-SDF represents the residue soluble fiber; P-SDF represents the peel soluble fiber; R-IDF represents the residue insoluble fiber; P-IDF represents the peel insoluble fiber.

FT-IR was carried out to analyze the spectroscopic features of different DF samples. As shown in Figure 2, there was a large absorption peak at 3200–3600 cm^−1^, which was the absorption of O-H stretching vibration band of cellulose and semi-fiber [27]. Compared with P-SDF and R-SDF, blue shifts were observed in both P-IDF and R-IDF, which may be due to the destruction of the hydrogen bonds formed in the hydroxyl groups of the polysaccharide during the ultrasonic treatment [28]. The breaking of hydrogen bonding could increase the hydrogen bonds between the citrus fiber and water, which might lead to increase the water-holding capacity of citrus fibers.

The broad peak appearing at 2800–3000 cm^−1^ was the stretching vibration of the C-H bond, which was a characteristic absorption peak of the polysaccharide methyl, in dictating that all four substances were polysaccharides [29]. All orange fibers had C-H stretching vibrations from methyl and methylene groups of polysaccharides at approximately 2927 cm^−1^. All samples had a characteristic peak at 1740–1760 cm^−1^ (C-O stretching of COOH), indicating that the ultrasound-assisted enzymatic method caused the hydrolysis of hemicellulose. In addition, the absorption peaks near 1646 cm^−1^ and 1419 cm^−1^ contained carbonyl C=O and carboxyl COOH, exhibiting that uranic acid was presented in DF. The strength of R-SDF intermolecular hydrogen bonds at the band of 1640 cm^−1^ was lower than that of the other three samples, which showed that ultrasonic cavitation might lead to the destruction of partial hydrogen bonds between R-SDF cellulose chains. This change could help citrus fiber hydrogen bonding loosen its internal structure and more fibers water hydrogen bonds when exposed to water.

In P-SDF and R-SDF, the peak intensity of 1016–1032 cm^−1^ was observed to decrease, indicating that the hydrogen bond between cellulose was broken after ultrasonic treatment, which is beneficial to the combination of pectin and cellulose. The absorption peak was at 867 cm^−1^ of carbohydrate characteristics, that is, the stretching vibration peak of β-glycosidic bond, and the peak values of R-IDF and P-IDF were more noticeable. The absorption peak of R-IDF at 1747 cm^−1^ was slightly stronger, which might contain more lignin. P-IDF had a strong absorption peak at 619 cm^−1^, which can probably be attributed to the presence of free alcohol or phenol. In addition, the four samples showed red shifts at some peak positions, indicating that the ultrasonic-assisted enzyme treatment destroyed the structure of organic molecules.

### 3.6. X-ray Diffractometry

**Figure 3:**
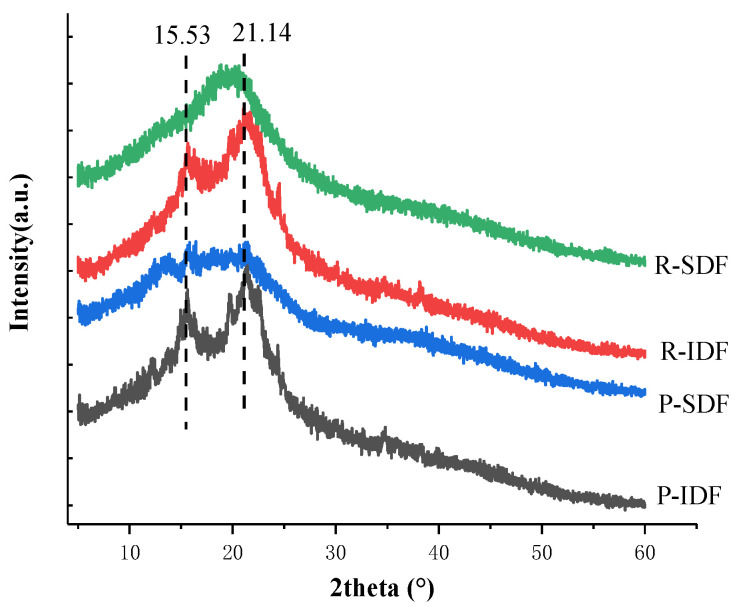
X-ray diffraction pattern of dietary fiber from R-SDF, P-IDF, P-SDF, R-IDF. R-SDF represents the residue soluble fiber; P-SDF represents the peel soluble fiber; R-IDF represents the residue insoluble fiber; P-IDF represents the peel insoluble fiber.

DF can be divided into ordered crystalline region and amorphous region [11]. The characteristic sharp front diffraction reflects the crystalline structure, and the diffusion diffraction reflects the amorphous structure. As shown in Figure 3, other samples had a crystalline peak at 15.53°2θ except the R-SDF sample. All the samples had the characteristic crystalline peaks at 21.14°2θ. For three kinds of DFs except R-SDF, the peak appeared at 15.53°2θ. Among them, the signal peak of 15.53° came from the crystal plane 101, the diffraction peak of 21.14°2θ corresponded to the crystal plane 002 [30]. The crystal plane diffraction peak showed that the four DFs had the coexistence of crystalline and amorphous states, which was a typical cellulose type I structure. The peak patterns of R-IDF and P-IDF were sharper, indicating higher crystallinity, which might be attributed to the decreased protein and lignin content [11]. The characteristic peaks of R-SDF and P-SDF were relatively gentle, and there was no very sharp and obvious crystallization peak [19], which might be because of SDF having a higher pectin content. There were no significant differences among the crystallinity values of P-IDF (42%) and R-IDF (44%), although the values of R-SDF (17%) and P-SDF (29%) were decreased using ultrasonic treatments. This latter observation was attributed to the fact that ultrasonic treatment leads to the disruption of cellulose chain and affects the molecular tissue of SDF [10]; however, the treatment might also have an effective on improving the swelling, water-holding and oil-holding capacities. 

### 3.7. Physicochemical and Functional Properties of SDFs and IDFs

#### 3.7.1. WHC, OHC, SC and CEC

Water-holding capacity (WHC), oil-holding capacity (OHC), swelling capacity (SC) and cation exchange capacity (CEC) are important indexes to evaluate the quality and physiological function of DF.

The WHC stands for the water retention capacity of the material; it can reduce dehydration and contraction of the product and promote digestion in the human body. As shown in Table 2, the WHC values of four samples ranged from 8.81 to 13.43 g/g. R-IDF (13.43 ± 0.20 g/g) had the best WHC, followed by R-SDF (11.97 ± 0.49 g/g), P-IDF (11.75 ± 0.35 g/g) and P-SDF (8.81 ± 0.36 g/g). The WHC values of DF samples extracted were higher than lemon (6.46 ± 0.79 g/g), orange (7.76 ± 0.81 g/g) and grapefruit seeds (7.06 ± 0.16 g/g) [31], which maybe because the special functional properties of ultrasound could result in a loose porous structure of DF samples. Furthermore, the WHC of extruded Liucheng sweet orange pomace of 6.73 g/g of orange dry sample was less than P-SDF (8.81 ± 0.36 g/g). The possible reason for this result may be that the high temperature in barrel destructed the water-holding capacity of the extrudate, which could not be well retained water within the DF [32]. This value of WHC indicated that ultrasound retains moisture better than extrusion.

In contrast to WHC, SC always depends on many factors such as network density, molecular size and the cellulose components of the fiber [19]. Table 2 shows that the SC of R-SDF (23.33 ± 1.15 mL/g) were significantly greater (*p* < 0.05) than those of P-SDF (19.33 ± 1.53 mL/g), R-IDF (18.44 ± 0.51 mL/g) and P-IDF (16.89 ± 0.38 mL/g). Soluble orange peel fibers had 4.83 and 6.28 mL/g SC values by steam explosion and dilute acid soaking, which were less than P-SDF and R-SDF [33]. This result may be because the ultrasound treatment can loosen the orange fibers’ inner structure and break hydrogen bonds, and the enzymatic treatment can further disrupt cellulose chains and remove some cellulose, which can be beneficial to the increase in swelling capacity.

Table 2 showed the OHC values of Newhall navel orange peel and residue DFs ranged between 1.26 and 2.08 g oil/g fiber. R-IDF (2.08 ± 0.21 g/g) was 65.08% greater than that of P-SDF (1.26 ± 0.05). In another study, the OHC values of various orange fibers ranged from 0.9 to 1.3 g/g [34]. Therefore, the OHC of R-SDF is within the reported ranges for the OHC values. The other three samples have higher OHC values, which may be related to their better structure and surface area. Previous studies have shown that fiber particles can absorb and combine oily components [35], which could make food have better palatability [27]. Therefore, four dietary fibers can become effective raw materials for the functional food industry. 

There are amino and hydroxyl groups in the molecular structure of dietary fiber. When these groups are exchanged with Zn^2+^ and Cu^2+^ plasma, the pH and osmotic pressure of human digestive tract can be changed, and the blood pressure can be lowered. As shown in Table 2, the CEC of R-SDF was the highest, followed by R-IDF and P-SDF, and P-IDF was the least. This might be due to the decomposition of some citrus pulp fibers caused by ultrasonic treatment, exposing more side groups such as hydroxyl, carboxyl and amino groups. DFs with high CEC will reduce the diffusion and absorption of lipids and cholesterol [2]. Besides, CEC can decrease the utilization of cholesterol in vivo [22].

#### 3.7.2. Cholesterol Adsorption Capacity (CAC)

**Figure 4:**
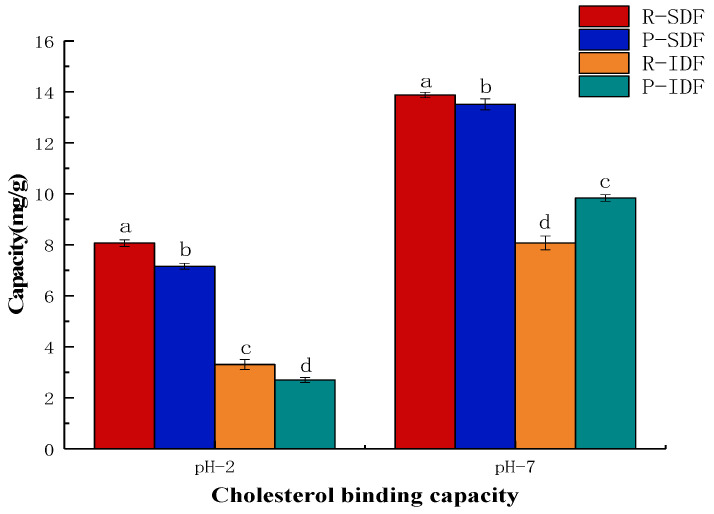
The CAC of R-IDF, P-IDF, R-SDF, P-SDF. R-SDF represents the residue soluble fiber; P-SDF represents the peel soluble fiber; R-IDF represents the residue insoluble fiber; P-IDF represents the peel insoluble fiber; values with different letters (a, b, c, d) for IDF or SDF are significantly different means (*p* < 0.05).

Cholesterol metabolism has a close relationship with the incidence of cardiovascular and cerebrovascular diseases, while DF can reduce the digestion and absorption of cholesterol and triglyceride by combining with food. Therefore, the CAC of Newhall navel orange peel and residue DFs was studied. The CACs of the DF samples are shown in Figure 4. The CACs of all the DF samples in simulated small intestinal pH value environment (pH = 7.0) were significantly (*p* < 0.05) stronger than those of under the condition of simulated stomach acidity (pH = 2.0), which is consistent with the behavior of bamboo shoot dietary fibers [36]. This may be because there is more H^+^ in an acidic environment, and the cholesterol in dietary fiber has a partial positive charge which produces repulsive force, thus reducing the adsorption force. Furthermore, the R-SDF showed the highest CAC in both pH = 2.0 (8.07 ± 0.13 mg/g) and pH = 7.0 (13.88 ± 0.10 mg/g), which might be due to the dietary fiber’s surface area. In addition, SDF exhibited a better healthy effect on reducing cholesterol than IDF in the cholesterol absorption, which is similar to previous studies [37,38].

#### 3.7.3. Nitrite Ion Absorption Capacity (NIAC)

**Figure 5:**
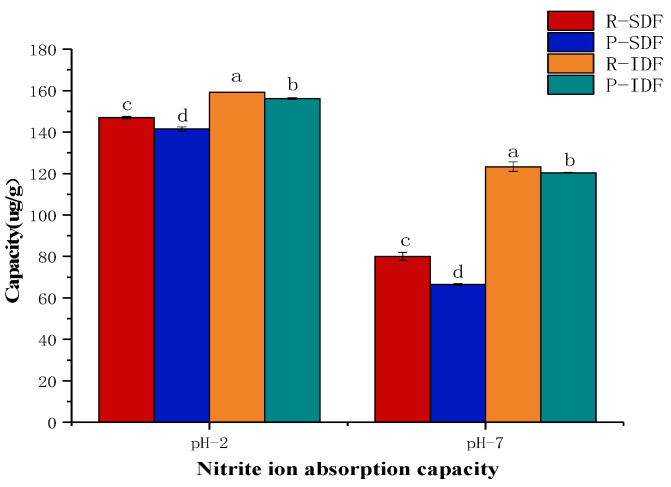
The NIAC of R-IDF, P-IDF, R-SDF, P-SDF. R-SDF represents the residue soluble fiber; P-SDF represents the peel soluble fiber; R-IDF represents the residue insoluble fiber; P-IDF represents the peel insoluble fiber; values with different letters (a, b, c, d) for IDF or SDF are significantly different (*p* < 0.05).

Nitrite is commonly used as a color protector for meat products. Nitrite reacts with secondary amines, tertiary amines and amines in food to form a strong carcinogen, N-nitrosamine [9]. The result of this study was presented in Figure 5. For all the dietary fiber samples had increased NIAC in pH = 2.0 environment compared with that at pH = 7.0, and IDFs had a higher NIAC than SDFs [9], which is owing to NO_2_^−^ combining with H^+^ to form HNO_2_, forming more nitrogen oxides. Among these DF samples, R-IDF revealed the highest NIAC in both pH = 2.0 (159.16 ± 0.16 μg/g) and pH = 7.0 (123.31 ± 0.18 μg/g), which might be due to the R-IDF sample having a better structure interaction with more nitrite ion [9]. In addition, R-SDF exhibited significantly higher NIAC than P-SDF in both pH = 2.0 and pH = 7.0 (146.99 ± 0.60 μg/g, 141.50 ± 0.99 μg/g, respectively) and pH = 7.0 (80.08 ± 1.97 μg/g, 66.49 ± 0.39 μg/g, respectively). The above results indicated that R-SDF had more functional groups that could interact with NO_2_^−^.

## 4. Discussion

In this study, the structural, physicochemical and functional properties of dietary fiber exacted from Newhall navel orange by-products were investigated. We accordingly found that SDF and IDF of orange residue in by-products extracted by ultrasound-assisted enzymatic method had the highest yield. Glucose and arabinose were the dominant monosaccharides of all DF samples in this study, and the content of monosaccharide in navel orange residue DFs was higher than navel peel DFs. Besides, the surface of P-IDF more than R-IDF has looser and more complicated structures, and R-SDF has a more complete surface than P-SDF. Among these four DF samples, R-IDF has the highest WHC (13.43 ± 0.20 g/g) and OHC (2.08 ± 0.21 g/g) values, whereas R-SDF has the highest SC (23.33 ± 1.15 mL/g) value and CEC (0.89 ± 0.01 mmol/g). The result of functional properties revealed that the R-SDF extracted from residue and peel had the highest CAC and NIAC. Considering the extraction efficiency and good quality retention, the R-SDF and R-IDF have the broadest market prospect for potential application, which could improve the economic efficiency and utilization rate of Newhall navel orange by-products.

## Figures and Tables

**Table 1:** The monosaccharide composition of R-SDF, P-SDF, R-IDF and P-IDF.

Sample	Mannose (mg/g)	Rhamnose (mg/g)	Galacturonic Acid (mg/g)	Glucose (mg/g)	Galactose (mg/g)	Xylose (mg/g)	Arabinose (mg/g)
R-SDF	0.44 ± 0.12 ^a^	0.58 ± 0.28 ^d^	1.41 ± 0.11 ^b^	20.51 ± 0.16 ^a^	3.72 ± 0.14 ^b^	0.16 ± 0.20 ^d^	3.18 ± 0.27 ^d^
P-SDF	0.75 ± 0.23 ^b^	1.71 ± 0.1 ^b^	1.57 ± 0.3 ^a^	20.16 ± 0.22 ^b^	1.79 ± 0.07 ^d^	0.26 ± 0.17 ^b^	3.52 ± 0.29 ^c^
R-IDF	0.33 ± 0.10 ^c^	1.40 ± 0.14 ^c^	1.19 ± 0.29 ^c^	8.18 ± 0.28 ^c^	5.12 ± 0.41 ^a^	0.22 ± 0.04 ^c^	11.31 ± 0.50 ^a^
P-IDF	0.36 ± 0.12 ^c^	2.27 ± 0.12 ^a^	1.58 ± 0.15 ^a^	5.94 ± 0.23 ^d^	3.53 ± 0.22 ^c^	1.28 ± 0.11 ^a^	8.19 ± 0.38 ^b^

R-SDF represents the residue soluble fiber; P-SDF represents the residue soluble fiber; R-IDF represents the residue insoluble fiber; P-IDF represents the residue insoluble fiber; data are expressed as the mean ± standard deviation (SD). Different letters (a, b, c, d) indicate significantly different means (*p* < 0.05).

**Table 2:** Physiochemical properties of R-SDF, P-SDF, R-IDF and P-IDF.

	R-SDF	P-SDF	R-IDF	P-IDF
WHC (g/g)	11.97 ± 0.49 ^b^	8.81 ± 0.36 ^c^	13.43 ± 0.20 ^a^	11.75 ± 0.35 ^b^
OHC (g/g)	1.34 ± 0.05 ^b^	1.26 ± 0.05 ^b^	2.08 ± 0.21 ^a^	1.36 ± 0.03 ^b^
SC (mL/g)	23.33 ± 1.15 ^a^	19.33 ± 1.53 ^b^	18.44 ± 0.51 ^bc^	16.89 ± 0.38 ^c^
CEC (mmol/g)	0.89 ± 0.01 ^a^	0.76 ± 0.02 ^c^	0.82 ± 0.02 ^b^	0.73 ± 0.03 ^c^

WHC, water-holding capacity; OHC, oil-holding capacity; SC, swelling capacity; CEC, Cation exchange capacity. R-SDF represents the residue soluble fiber; P-SDF represents the peel soluble fiber; R-IDF represents the residue insoluble fiber; P-IDF represents the peel insoluble fiber; data are expressed as the mean ± standard deviation (SD). Different letters (a, b, c) indicate significantly different means (*p* < 0.05).
